# Quantifying the Effects of Different Treadmill Training Speeds and Durations on the Health of Rat Knee Joints

**DOI:** 10.1186/s40798-018-0127-2

**Published:** 2018-04-02

**Authors:** Jaqueline Lourdes Rios, Kevin Rudi Boldt, James William Mather, Ruth Anne Seerattan, David Arthur Hart, Walter Herzog

**Affiliations:** 1 Faculty of Kinesiology, Human Performance Laboratory, University of Calgary, 2500 University Drive NW, Calgary, AB T2N 1N4 Canada; 2 McCaig Institute for Bone and Joint Health, University of Calgary, Calgary, AB Canada; 3 CAPES Foundation, Brasilia, DF Brazil

**Keywords:** Osteoarthritis, Cyclical loading, Aerobic exercise, Joint health, Histology, Animal model

## Abstract

**Background:**

Walking and running provide cyclical loading to the knee which is thought essential for joint health within a physiological window. However, exercising outside the physiological window, e.g. excessive cyclical loading, may produce loading conditions that could be detrimental to joint health and lead to injury and, ultimately, osteoarthritis. The purpose of this study was to assess the effects of a stepwise increase in speed and duration of treadmill training on knee joint integrity and to identify the potential threshold for joint damage.

**Methods:**

Twenty-four Sprague-Dawley rats were randomized into four groups: no exercise, moderate duration, high duration, and extra high duration treadmill exercise. The treadmill training consisted of a 12-week progressive program. Following the intervention period, histologic serial sections of the left knee were graded using a modified Mankin Histology Scoring System. Mechanical testing of the tibial plateau cartilage and RT-qPCR analysis of mRNA from the fat pad, patellar tendon, and synovium were performed for the right knee. Kruskal-Wallis testing was used to assess differences between groups for all variables.

**Results:**

There were no differences in cartilage integrity or mechanical properties between groups and no differences in mRNA from the fat pad and patellar tendon. However, COX-2 mRNA levels in the synovium were lower for all animals in the exercise intervention groups compared to those in the no exercise group.

**Conclusions:**

Therefore, these exercise protocols did not exceed the joint physiological window and can likely be used safely in aerobic exercise intervention studies without affecting knee joint health.

## Key Points

- A stepwise increase in the speed and duration of exercise did not lead to osteoarthritis-like changes in the knee.
- Chronic exercise appeared to produce a protective effect on the knee.
- Working within an optimal physiological exercise window is beneficial for joint health across the life span.

## Background

Exercise has been used to promote health and fitness as far back as 2500 BC [1]. However, in the past years, exercise has been postulated to act like a drug [2] and, as such, provides advantages and risks to an individual’s health and fitness. Specifically, aerobic exercise has been shown to lead to increased bone mineral density, insulin sensitivity, high-density lipoprotein cholesterol levels, resting and maximal stroke volume, maximal oxygen uptake, and basal metabolism [3, 4]. Exercise has also been shown to reduce body fat, fasting blood insulin levels, low-density lipoprotein cholesterol levels, resting heart rate, and systolic and diastolic blood pressure [3, 4]. However, the effects of exercise on joint health remain controversial [5–8].

In a prospective survey, aimed at examining the relationship of self-reported physical activity and physician-diagnosed osteoarthritis (OA) from 1970 to 1995, a positive association between OA incidence and physical activity was found. Running was suggested to lead to an increased risk for developing OA in some studies [8], but not in others [5–7]. Chakravarty et al. [5] did not find an increased risk for knee OA in masters runners compared to controls over an 18-year period [5], and Miller et al. [7] suggested that running does not increase the risk for knee OA compared to walking. However, Cheng et al. [8] reported a positive association between running more than 32 km per week and clinician-diagnosed knee OA.

If exercise is thought to act like a drug preventing joint disease, the dosage may be critical to its success. It has been shown that moderate exercise, such as walking and running, exerts added loading to the knee joint, and cyclical loading is thought to be vital for maintaining cartilage integrity/homeostasis and healthy joints [9–11]. In contrast, in vivo and vitro studies with excessive repetitive joint loading have been implicated with the development of OA [12, 13]. Joints are thought to be designed to operate within a “physiological window” to maintain proper function and allow for positive adaptations [12]. Loading outside this window may put the joint, and specifically the cartilage, at risk for degeneration [14, 15].

In rodent model of exercise, for example, Galois et al. [16] suggested that different levels of treadmill training may have different influences on the severity of chondral lesions in anterior cruciate ligament transection (ACLT) model of osteoarthritis in Wistar rats; while slight to moderate levels seemed to be beneficial to the knee cartilage health, more strenuous exercise may be detrimental. Controversially, Yang et al. [17] showed that treadmill exercise up to 1 h per day, 5 days a week, for 8 weeks, in Sprague-Dawley rat model of monosodium iodoacetate-induced OA, has a chondroprotective effect, and this effect was more prominent in rats that fulfill in three times per day the 1 h treadmill exercise, suggesting that an adaptive phase training may be an important factor in protecting the knee joint from OA-like changes.

Wistar rats, without previous lesion in the knee, have also been randomly assigned to a sedentary control group, a low-intensity running, a medium-intensity running, and a high-intensity running [18]. As a result, rats in the high-intensity running demonstrated OA-like changes in their knees, while rats in the other running groups did not. Moreover, in a study in rodent models where the animals were allowed minimum [19] or not allowed an adaptive phase [18], they have demonstrated OA-like changes in their knees. However, it is unknown how much exercise is too little or too much. Therefore, the purpose of this study was to determine the effects of a stepwise increase in speed and duration of treadmill training on knee joint integrity and to identify the potential treadmill training threshold for the development of joint damage in the rat knee. We hypothesized that excessive chronic treadmill training leads to the development of OA-like changes in the knee, while moderate levels of treadmill training maintain cartilage integrity and induce positive adaptive responses. The key outcome measures were joint integrity, body composition, gene expression patterns, and blood-based and synovial fluid-based biomarkers.

## Methods

The aim of this study was to determine the effects of a stepwise increase in speed and duration of treadmill training on knee joint integrity and to identify the potential treadmill training threshold for the development of joint damage in the rat knee.

### Animals

Twenty-four 10- to 14-week-old male Sprague-Dawley rats were housed individually and randomized into four groups: moderate duration exercise (MD, *n* = 6), high duration exercise (HD, *n* = 6), extra high duration exercise (EHD, *n* = 6), or no exercise (control, *n* = 6). Rats were fed (ad libitum) a standard chow diet (Diet #5001, Lab Diet, USA). A minimum sample size of five rats per group is based on the ability to detect a minimal meaningful difference in histological scoring of the knee joint to provide an *α* = 0.05 and a power of 80%. Calculation of sample size was performed using G*Power Software (version 3.0.10, Germany) [20]. Data for sample size calculations were obtained from a previous study [21]. All experiments were approved by the University of Calgary Life and Environmental Sciences Animal Care Committee, and all methods were conducted in accordance with the animal welfare regulations and guidelines at the University of Calgary.

### Exercise Training Protocol

Following 1 week of acclimatization to the housing environment, rats were exposed to their respective exercise programs (see Table 1 for details of the exercise programs) on a Columbus Instruments Exer-3R treadmill (Columbus, OH, USA) for 12 weeks. The moderate duration group progressively built up to 30 min of treadmill training each day, five times per week at 25 m/min. The high duration group built up to 60 min of treadmill training per day for 5 days per week at 25 m/min. Rats in the extra high duration protocol reached 60-min training sessions 7 days per week at 25 m/min. In weeks 10, 11, and 12, these rats trained twice, three times, and four times for 1 h daily, respectively. This last training intervention has previously been used to elicit overtraining in Wistar rats [22]. Animals in the control group were placed on the treadmill 5 days a week and completed 15 min of exercise at 10 m/min once per week. This was done to account for the stress of handling and avoiding confound results. A shock grid at the back of the treadmill was used to prevent animals from falling behind the pace of the treadmill.

**Table 1:** Twelve-week treadmill training programs for groups of rats

Week	Speed (m/min)	Moderate duration			High duration			Extra high duration		
		Session/week	Session/day	Duration (min)	Session/week	Session/day	Duration (min)	Session/week	Session/day	Duration (min)
1	0	5	1	10	5	1	10	5	1	10
2	15	5	1	20	5	1	20	7	1	20
3	20	5	1	30	5	1	30	7	1	30
4	22.5	5	1	30	5	1	45	7	1	45
5	25	5	1	30	5	1	60	7	1	60
6–9	25	5	1	30	5	1	60	7	1	60
10	25	5	1	30	5	1	60	7	2	60
11	25	5	1	30	5	1	60	7	3	60
12	25	5	1	30	5	1	60	7	4	60
Total traveled distance		37.9 km			69.6 km			162.8 km		
		23.6 miles			43.2 miles			101.2 miles		

*Week 1:* familiarization week

Note that the extra high duration group had an increase in session number per day on weeks 10, 11, and 12. Rats in the control group walked per 15 min once a week at 10 m/min. The experimental week for rats in the moderate duration, and high duration groups, consisted of five consecutive days of training sessions, with 24 h of recovery between sessions, followed by 2 days of recovery. The experimental week for rats in the extra high duration group consisted of daily training sessions with 24 h recovery between sessions from week 2 to week 9. On weeks 10, 11, and 12, they had 6, 4, and 2 h recovery, respectively, between training sessions on the same day

### Body Composition

Body mass was measured at the beginning of each week. Body mass for each animal was normalized to that of week 1 (familiarization week) and was expressed as the percent increase in body mass from that initial value. One week after completing the 12-week training protocol, and immediately prior to sacrifice, rats were lightly anesthetized with isoflurane and body composition was measured using dual X-ray absorptiometry (DXA) with software for small animals (Hologic ODR 4500; Hologic, MA, USA). An average of three scans for each animal was used for analysis.

### Blood Analysis

Following 12 h of fasting, rats were anesthetized with isoflurane and a blood sample was collected by cardiac puncture. Blood was centrifuged at 3000 rpm for 15 min at 4 °C and serum stored in aliquots at − 80 °C until analyzed. Rats were sacrificed by heart excision. Serum cytokines and adipokines were quantified using a Rat 27 Multiplex Discovery Assay with Luminex® xMAP technology (eotaxin, EGF, fractalkine, IL-1α, IL-1β, IL-2, IL-4, IL-5, IL-6, IL-10, IL-12(p70), IL-13, IL-17A, IL-18, IP-10/CXCL10, GRO/KC, IFN-γ, TNF-α, G-CSF, GM-CSF, MCP-1, leptin, LIX, MIP-1α, MIP-2, RANTES, VEGF; Eve Technologies, Calgary, AB, Canada).

### Knee Joints

Knee joints were collected from both hind limbs. The left knee was harvested by cutting the femur and tibia/fibula 2 cm above and below the joint line. Excess muscles were removed, and joints were fixed in a 10% neutral buffered formalin solution (Thermo Fisher Scientific, MA, USA) for 14 days at room temperature. Knees were then decalcified at room temperature, using Cal-Ex II solution (10% formic acid in formaldehyde, Thermo Fisher Scientific). The decalcification solution was changed daily. The end of decalcification was determined by chemical testing with a 5% ammonium oxalate solution (Thermo Fisher Scientific) until no precipitate was detected for 5 days (on average 21 days). Samples were then processed using an automatic paraffin processor (Leica TP 1020, Leica Microsystems Inc., Concord, Ontario, Canada). They were dehydrated in a graded series of alcohols, cleared in xylene, and infiltrated with 50% Paraplast X-TRA® wax (Thermo Fisher Scientific) and 50% Paraplast Plus® wax (Thermo Fisher Scientific). Further, the left knee joints were embedded in paraffin wax and stored at room temperature until sectioning. Serial, sagittal plane sections of 10-μm thickness were obtained using a Leica RM 2165 microtome (Leica Biosystems, Nussloch, Germany). Sections were mounted onto Super Frost plus slides (Thermo Fisher Scientific) and allowed to dry at 40 °C for 7 days. Alternate slides were stained sequentially with hematoxylin, fast green, and safranin-O (Thermo Fisher Scientific) using an auto stainer (Leica ST 5010, Leica Biosystems). Sections were then dehydrated in a graded series of alcohols, cleared in xylene, and mounted with Cytoseal 60 mounting media (Thermo Fisher Scientific) using an auto cover slipper (Leica CV 5030, Leica Biosystems). Slides were dried at room temperature for 7 days before being evaluated using a light microscope (Zeiss Axiostar plus, Carl Zeiss Inc., Ontario, Canada). Two independent graders scored all histological sections in a blinded manner using a modified Mankin Histology Scoring System [23]. Osteoarthritis Research Society International (OARSI) histologic [24] subscores for bone changes, synovial thickening, and meniscus were also determined for each joint. The total modified Mankin score for each animal represents the sum of all Mankin scores and OARSI subscores. Tibial and femoral cartilage thickness were determined from histological slides.

The right knee joints were opened to collect the synovium, menisci, fat pad, and patellar tendon. The tissues were snap-frozen in liquid nitrogen and stored at − 80 °C for RNA isolation and subsequent RT-qPCR analysis. Genes analyzed for the patellar tendon and synovium were Col-1, Col-3, iNOS, COX-2, IGF-1, IL-1, IL-6, and TGF-β. Genes analyzed for the fat pad were iNOS, PPAR-γ, COX-2, IL-1B, IP-10, leptin, TF, TFPI, and VEGF. Genes analyzed for the menisci were Col-1, Col-3, iNOS, PPAR-γ, COX-2, IGF-1, IL-1, IL-6, TGF-β, IP-10, leptin, TF, TFPI, VEGF, and PRG4 (see Table 2 for details). Synovial fluid was also collected from the right knee shortly after sacrifice using the Whatman chromatography paper method [25]. Samples were weighed, diluted 1:50, and stored at 4 °C overnight. After 24 h, samples were centrifuged and stored at − 80 °C until analysis. Synovial fluid cytokines and adipokines were quantified using a Rat 27 Multiplex Discovery Assay with Luminex® xMAP technology (eotaxin, EGF, fractalkine, IL-1α, IL-1β, IL-2, IL-4, IL-5, IL-6, IL-10, IL-12(p70), IL-13, IL-17A, IL-18, IP-10/CXCL10, GRO/KC, IFN-γ, TNF-α, G-CSF, GM-CSF, MCP-1, leptin, LIX, MIP-1α, MIP-2, RANTES, VEGF; Eve Technologies).

**Table 2:** Primer sequence used for RT-qPCR

PRIMER	Forward sequence	Reverse sequence	Source
18S (human)	TGG TCG CTC GCT CCT CTC C	CGC CTG CTG CCT TCC TTG G	NR_003286
Col I	CAG ACG GGA GTT TCA CCT C	GAC ATG TAG ACT CTT TGC GGC	J04464
Col III	CTA ACC AAG GCT GCA AGA TG	ATC TGT CCA CCA GTG CTT CC	AJ005395
COX-2	CAG TAC ACT ACA TCC TGA CC	CGT CAA CAC GTA TCT CAT GG	S67722
IGF-1	GCA TTG TGG ATG AGT GTT GC	GGT CTT GTT TCC TGC ACT TC	X06043
IL-1A	CAG GCA TCC TCA GCA GCA GA	GGC TCC TAA GAA CAA GAA TG	NM_017019
IL-1b	AAC CTG CTG GTG TGT GAC GTT C	CAG CAC GAG GCT TTT TTG TTG T	NM_008361
IL-6	TCA CAG AAG GAG TGG CTA AG	ACC ACA GTG AGG AAT GTC CA	NM_012589
iNOS	AAG GCA CAA GAC TCT GAC AC	GGA TCG CAC TTC TGT CTC TC	AB250951
IP10	CAA GGC TTC CCA ATT CTC	ACC TGG ACT GCA TTT GA	U22520
Leptin	CCT GTG GCT TTG GTC CTA TCT G	CTG CTC AAA GCC ACC ACC TCT G	NM_013076
PPAR-γ	CTT GGC CAT ATT TAT AGC TGT CAT TAT T	TGT CCT CGA TGG GCT TCA C	NM_013124
PRG4	AGG GCG TTG CAT CCA AGA A	ATA ATC ACA GTT GCA GGT GGC	NM_001105962
TF	GAC AAT CTT GGA GTG GCA AC	GCT TCA TAG GTC CAG TTC AC	NM_013057
TFPI	CCG AGG AAG CTA TGT GTA AG	AGC CAG TGT AGG TGA AGG TC	NM_173141
TGFB	ACG GCA GCT GTA CAT TGA CT	CAG GAG CGC ACG ATC ATG TT	NM_021578
VEGF	TTA CTG CTG TAC CTC CAC CA	ATA GTG CAG TTG CTC TCC GA	NM_031836

The right tibia was stored at − 20 °C until assessment of the biomechanical properties of cartilage was performed. A spherical indenter (*r* = 175 ± 2.5 μm) made of a stainless steel 316 L shaft and a spherical glass bead was installed under the multiaxial load cell (force resolution: Fz = 3.5 mN and Fx = Fy = 2.5 mN) of a three-axis mechanical tester (Mach-1 v500css, Biomomentum, QC, Canada). The tibia was fixed in a sample holder using dental cement. The sample was then immersed into a testing chamber that contained PBS and was equipped with a camera registration system (Biomomentum). A position grid was superimposed on the image of the tibia articular surface for a mechanically controlled surface mapping [26]. Stress relaxation tests for cartilage properties were performed on 11 sites each for the medial and lateral tibial plateau using the automated mapping system, and Young’s modulus were calculated for each test site.

### Statistical Analysis

Non-parametric Kruskal-Wallis testing was used to determine differences between the four animal groups for all variables (RT-qPCR analyses, body fat, body mass, cartilage thickness, Mankin/OARSI score, equilibrium Young’s modulus, and protein assays). If significant (*p* < 0.05), post hoc testing using the Mann-Whitney *U* test was used to indicate differences between groups. Further, estimates of effect size were calculated using the ANOVA univariate approach on SPSS (version 22). Partial eta squared (partial *η*^2^) were reported to provide small (partial *η*^2^ = 0.01), medium (partial *η*^2^ = 0.06), and large (partial *η*^2^ = 0.14) effects [27].

## Results

### Training Distances

**Fig. 1:**
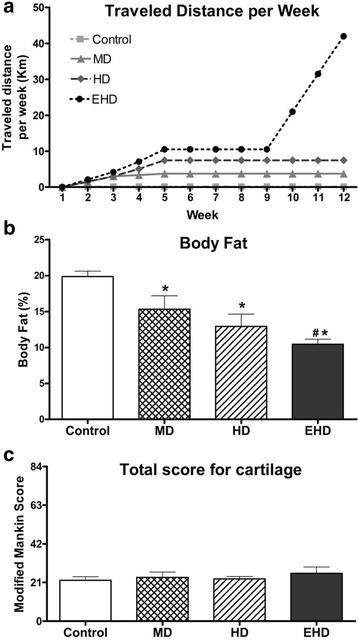
**a** Travel distance per week per group. Notice, the control group was exposed to a constant amount of exercise throughout the protocol, 150 m per week. For the other groups, there was a gradual increase in the amount of exercise from week 2 to week 5. From week 5 to the end of the intervention period, the levels of exercise were constant for rats in the MD and HD groups. For rats on the EHD group, the exercise distance doubled, tripled, and quadrupled in weeks 10, 11, and 12 compared to weeks 5–9, respectively. **b** Body fat. % body fat measured in the end of the experimental protocol. **c** Total modified Mankin scores: No significant differences were found between groups. MD moderate duration exercise, HD high duration exercise, EHD extra high duration exercise. * indicates significant difference when compared to control group. # indicates significant difference when compared to moderate duration group

Rats in the control group traveled a distance of 150 m per week from week 2 to week 12, with a total distance of 1.65 km at the end of the experimental protocol. Rats in the MD and HD groups had a gradual increase in speed and duration from week 2 to week 5 (4 weeks of adaptation), and then, they ran in a constant speed and duration from week 6 to week 12, leading to a total distance traveled of 37.9 and 69.9 km, respectively (Fig. 1, Table 1). Rats in the EHD group have a stepwise increase in distance traveled from week 2 to week 5, then a constant distance traveled from week 6 to week 9, and another stepwise increase in distance traveled from week 10 to week 12, leading to a total distance traveled of 162.8 km by the end of the exercise protocol.

### Body Composition

Rats in all groups exhibited a gradual increase in body mass during the intervention period. However, body fat was significantly reduced by 4.5% (*p* = 0.046), 6.9% (*p* = 0.032), and 9.4% (*p* = 0.003) in the MD, HD, and EHD group rats, respectively, compared to that in the control group rats at the end of the exercise intervention period. Body fat was also significantly reduced by 4.9% (*p* = 0.025) in the EHD rats compared to that in the MD group rats (Fig. 1b, Table 3).

**Table 3:** Estimates of effect size calculated using ANOVA univariate approach. Cohen [27] has provided point of reference to define small (partial *η*^2^ = 0.01), medium (partial *η*^2^ = 0.06), and large (partial *η*^2^ = 0.14) effects

Variable	Partial *η*^2^
% body fat	0.578
Knee joint	
Total score	0.062
MTP	0.025
MFC	0.111
LTP	0.033
LFC	0.114
Pat	0.209
Grv	0.255
Synovium	0.128
Menisci	0.075
Bone	0.197
Cartilage thickness	
MFC-ant	0.206
MFC-post	0.154
LFC-ant	0.131
LFC-post	0.143
MTP	0.204
LTP	0.177
Synovium: COX-2	0.679

*MTP* medial tibial plateau, *MFC* medial femoral condyle, *LTP* lateral tibial plateau, *LFC* lateral femoral condyle, *Pat* patella, *Grv* grove, *ant* anterior, *post* posterior

### Knee Joint Integrity and Inflammatory Markers

**Fig. 2:**
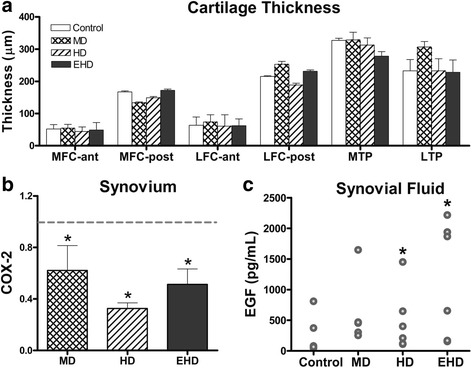
**a** Cartilage thicknesses across six sites on the knee joint measured through histology. No significant differences were found between groups. **b** COX-2 mRNA levels for the synovium expressed as fold change from control group (dashed gray line). **c** EGF exhibiting significant differences between groups for synovial fluid; 27 proteins were analyzed with a Luminex-based protein assay. MD moderate duration exercise, HD high duration exercise, EHD extra high duration exercise, MFC-ant anterior medial femoral condyle, MFC-post posterior medial femoral condyle, LFC-ant anterior lateral femoral condyle, LFC-post posterior lateral femoral condyle. * indicates a significant difference when compared to control group values

There were no differences in cartilage integrity (Fig. 1c, Table 3), mechanical properties, and cartilage thicknesses (Fig. 2a, Table 3) between groups across all joint locations. There were also no differences between groups in gene expression levels for the fat pad, patellar tendon, and menisci. Cyclooxygenase 2 (COX-2) levels in the synovium were lower for the three exercise group animals than those in the control group animals (*p* = 0.010) (Fig. 2b). Epidermal growth factor (EGF) levels in the synovial fluid were significantly greater in EHD and HD group animals than those obtained in the control group animals (*p* = 0.039, *p* = 0.014, respectively; Table 4, Fig. 2c). Serum interleukin-12 (IL-12) levels were significantly higher for the EHD group animals than for the control (*p* = 0.015) group and the HD (*p* = 0.008) group animals (Table 4).

**Table 4:** Kruskal-Wallis *p* values for serum and synovial fluid for the 27 protein analyzed using Luminex-based protein assay after 12 weeks on exercise

Protein	Serum	SF	Protein	Serum	SF
G-CSF	0.310	0.246	IL-5	0.124	0.542
Eotaxin	0.293	0.65	IL-17A	0.467	0.051
IL-1α	0.742	0.216	IL-18	0.960	0.317
Leptin	0.130	0.209	MCP-1	0.666	0.151
MIP-1α	0.647	0.203	IP-10	0.820	0.435
IL-4	0.569	0.181	VEGF	0.398	0.486
IL-1β	0.640	0.255	Fractalkine	0.475	0.111
IL-2	0.364	0.116	LIX	1.000	0.715
IL-6	0.352	0.222	MIP-2	0.588	*
EGF	0.374	0.027**	TNFα	0.080	0.166
IL-13	0.229	0.168	RANTES	0.983	0.961
IL-10	0.499	0.503	GM-CSF	*	*
IL-12	0.014**	0.487	GRO/KC	*	*
IFNy	0.203	0.483			

*No statistics were computed due to extrapolated values and/or values out of validated range

**Indicates *p* < 0.05.

*SF*: synovial fluid

## Discussion

Inactivity is the fourth leading risk factor for global mortality [28]. A physically inactive lifestyle is associated with the development of non-communicable diseases [29], including OA [30]. In contrast, exercise is reported to be a strong factor in health promotion and the prevention or delay of many non-communicable diseases [29]. However, poor health outcomes have been reported in athletes exposed to extra high levels of exercise [31, 32], and it has been reported that top-level athletes have an increased risk of developing knee OA [33]. Consequently, exercise can be beneficial or detrimental to the general health. Mechanical loading of joints due to exercise may be minimal but sufficient to maintain tissue homeostasis, it may be within the optimal physiological window and help maintain tissue homeostasis and produce positive tissue adaptations, or it may be strenuous, thus exceeding the physiological window and lead to disruptions of tissue homeostasis, leading to joint disease. In this study, we investigated the effects of a stepwise increase in speed and duration of treadmill training on knee joint health in Sprague-Dawley rats.

We randomized 24 male Sprague-Dawley rats into four groups: moderate duration exercise (MD), high duration exercise (HD), extra high duration exercise (EHD), or no exercise (control). We did not detect changes in cartilage structure, mechanical properties, or thickness between groups and across joint locations. Therefore, the speed and duration of even the most strenuous treadmill exercise protocol used here (up to 4 h of exercise daily) was not detrimental to the knee joint cartilage in these animals. Thus, the load applied to the knee through the exercise intervention protocol provided here was likely within the physiologic loading window that has been suggested to allow for adaptation, remodeling, and proper functioning of cells and tissues [9–12, 15].

An aggressive endurance exercise protocol in 16- to 18-week-old Wistar rats has been shown to lead to OA-like changes in the knee [19]. These rats were allowed 1 week of familiarization to the exercise protocol, followed by exercising twice a day, 5 days per week, for 3 and 6 weeks, for a total of 30 and 55 km of distance traveled [19]. In our study, rats were allowed a prolonged adaptation period and were introduced gradually to the increasing speed and duration of the exercise protocol, a factor which may be critical for joint health outcomes. The gradual increase in speed and duration of the exercise sessions may have allowed the cartilage to adjust gradually to the increasing load requirements, operating within the physiological window. The adaptive training phase in our study consisted of 4 weeks, and has been suggested to trigger increases in fitness and health in Wistar rats [22], and may be an important factor in the protection of the knee joint. It has been shown that a gradual increase in running volume in Wistar rats results in bone and cartilage remodeling by reducing catabolic genes and increasing aggrecan expression [34]. However, to our knowledge, there is no study systematically investigating adaptive training phase in order to protect the knee joint, and this is a limitation of our study. Additionally, in the present study, rats in the EHD group doubled, tripled, and quadrupled the amount of exercise in weeks 10, 11, and 12, respectively, relative to weeks 5–9. If these levels of exercise were sustained for an extended period, they may cause damage to the knee. Additionally, the physiological window for knee health is thought to be highly individual and may be influenced by genetics, sex, lifetime loading history, presence of prior injury/scar tissue, systemic pathology, and local anatomy [15].

Rats from all groups exhibited a gradual increase in body mass during the exercise intervention period. This increase was consistent with the expected increase in body mass of laboratory rats with age [35]. However, the MD, HD, and EHD group rats were leaner than the control group rats, and the EHD group rats were leaner than the MD group rats at the end of the intervention period. Body fat has been shown to decrease with aerobic exercise in humans [36] and mice [37]. Since body fat was reduced in the exercise group animals, but body weight was similar across all groups, the lean body mass (muscle mass) must have increased in the exercise group animals compared to that in the control group animals (not evaluated in our study). Results similar to ours have been reported for human studies where strength and endurance exercise did not produce differences in body mass but resulted in a reduction in body fat for individuals in an exercise program compared to individuals in a non-exercising group [38]. A reduction in body fat is considered a positive outcome for overall health and has been shown to reduce risks for diabetes, cardiovascular disease, metabolic syndrome, and knee OA [39–42].

Exercise induces multiple biochemical changes that may affect gene expression [43, 44]. In the present study, markers for oxidative stress (iNOS), collagen (Col-I and Col-III), pro-inflammation (IL-1β, COX-2, IL-6, leptin, IGF-1, IP-10), and anti-inflammation (TF, TFPI, VEGGF, TGF-β) were evaluated for the knee joint tissues. Gene expression in the fat pad, patellar tendon, and menisci were similar across all groups. However, COX-2 mRNA levels in the synovium were reduced for all animals in exercise groups compared to those in the animals in the control group. A reduction in COX-2 expression is thought to be positive for the joint environment, since drugs that inhibit COX-2 have been associated with a reduction of OA-like histological changes and suppressed chondrocyte apoptosis [45, 46]. Indeed**,** it has been demonstrated that exercise is a robust approach to preserve healthy cartilage [47]. Specifically, low intensity treadmill walking for 2, 5, or 15 days has been demonstrated to regulate metabolic responses at the cellular and systemic level, protecting cartilage against OA by altering gene expression of markers involved in OA onset [47]. Our findings indicate that all levels of exercise used in our study led to a reduction in COX-2 mRNA in the synovium, suggesting a potential protective effect of exercise for the knee, even in the lowest end highest exercise programs. It is important to highlight that at the end of the 12-week treadmill training, many molecular changes may have occurred to adapt to the new loading conditions and had already plateaued and normalized to the new exercise threshold by the time tissue was taken. Additionally, we did not study the protein and activity levels; this needs to be done in future studies.

Synovial fluid EGF levels were significantly higher in the EHD and HD group animals than those in the control group animals. It has been suggested that EGF is produced by the synovium in the initial stages of rheumatoid arthritis [48] and that activation of EGF receptor signaling may be a causal factor in OA [49, 50]. However, more recent studies indicated anabolic effects of EGF receptor signal activation in articular cartilage, suggesting that EGF may promote the expansion and/or activity of an endogenous EGF receptor responsive cell population within the articular cartilage [51]. Moreover, the EGF receptor is an important signaling molecule in bone development and remodeling and plays an anabolic role in bone metabolism [52]. Overall, the finding of elevated synovial fluid EGF levels in the EHD and HD group animals in the absence of OA-like changes suggests that EGF was not detrimental to knee joint health.

Serum IL-12 levels were significantly higher for the EHD group compared to those for the control group and HD group rats. This result agrees with the findings from in vitro studies. Exercise has been reported to increase IL-12 production by macrophages following lipopolysaccharide stimulation [53]. IL-12 is believed to be essential for the clearance of bacterial infections [54] and is thought to be an important pathogenetic factor in Th1-type-mediated autoimmune disease. IL-12-deficient mice have been found to be resistant to collagen-induced inflammatory arthritis [55], while transgenic over-expression of IL-12 exacerbates the course of this disease [56]. However, the role of IL-12 in maintaining knee joint integrity, and its role in the pathogenesis of osteoarthritis, is poorly understood. It should be noted that the over-expression of serum IL-12 may represent responses to exercise unrelated to the knee joint, as serum may represent input from a number of sources, i.e. muscle and vasculature. However, in combination with the other findings in our study, IL-12 may be playing a protective role for knee joint cartilage. Further mechanistic studies are required to elucidate the role of IL-12 in this model of exercise-induced changes in knee joint health, as well as follow the EHD rats for longer period of time than the 12 weeks of this protocol.

## Conclusions

In summary, a stepwise increase in the speed and duration of a 12-week chronic treadmill exercise program did not lead to OA-like changes in the rat knee but appeared to produce a potential protective effect through a reduction in COX-2 mRNA levels in the synovium. Further studies aimed at elucidating the preventive and potentially harmful effects of repetitive chronic exercise need to be performed to better understand the effects of joint loading on joint health above or below the physiological window. Working within an optimal physiological exercise window is beneficial for general and for joint health across the life span. It is also important in the context of recreational and elite sports, where the optimal window may be altered following joint injury or disease and may thus affect the return to sport.

## Abbreviations

BC: Before Christ
Col-1: Type I collagen
Col-3: Type II collagen
COX-2: Cyclooxygenase-2
DXA: Dual X-ray absorptiometry
EGF: Epidermal growth factor
EHD: Extra high duration
G-CSF: Granulocyte colony-stimulating factor
GM-CSF: Granulocyte macrophage colony-stimulating factor
GRO/KC: CXCL1 chemokine, growth-related oncogene keratinocyte chemoattractant
HD: High duration
IFN-γ: Interferon gamma
IGF-1: Insulin-like growth factor 1
IL: Interleukin
iNOS: Inducible nitric oxide synthase
IP-10/CXCL10: Interferon gamma-induced protein 10
LIX: CXCL5 chemokine
MCP-1: Macrophage chemoattractant protein-1
MD: Moderate duration
MIP: Macrophage inflammatory protein
mRNA: Messenger ribonucleic acid
OA: Osteoarthritis
OARSI: Osteoarthritis Society International (Histology Scoring System)
PPAR-γ: Peroxisome proliferator-activated receptor gamma
RANTES: Regulated on activation, normal T cell expressed and secreted
RNA: Ribonucleic acid
RT-qPCR: Real-time quantitative polymerase chain reaction
TF: Tissue factor
TFPI: Tissue factor pathway inhibitor
TGF-β: Transforming growth factor-beta
Th1: Type 1 T helper cells
TNF-α: Tumor necrosis factor alpha
VEGF: Vascular endothelial growth factor
